# Hepatocyte Growth Factor Differentially Modulates Oral Microbiota in Early vs. Late Experimental Periodontitis

**DOI:** 10.3390/biology14101393

**Published:** 2025-10-11

**Authors:** Ruotong Ji, Xiaomin Zhao, Zhen Chen, Yifei Ge, Zhicong Wu, Xinhong Wang

**Affiliations:** 1 School of Stomatology, Guangdong Engineering Research Center of Oral Restoration and Reconstruction & Guangzhou Key Laboratory of Basic and Applied Research of Oral Regenerative Medicine, Guangzhou Medical University, Guangzhou 510180, China; jrtaimayday@163.com (R.J.);; 2 Department of Periodontology, Jinan Key Laboratory of Oral Tissue Regeneration, Binzhou Medical College, Yantai 264000, China

**Keywords:** periodontitis, hepatocyte growth factor, microbiota

## Abstract

**Simple Summary:**

Periodontitis is a chronic disease caused by an imbalance in oral bacteria. We previously found that hepatocyte growth factor (HGF) can protect against early-stage disease but worsen it later. In this study, we examined how HGF affects bacteria attached to ligatures placed around teeth in mice with periodontitis. We compared normal mice and mice with high HGF levels, analyzing bacterial composition, inflammatory markers, and bone metabolism indicators at different disease stages. HGF significantly changed bacterial diversity and composition over time. Some bacteria, like *Lactobacillus*, showed opposite patterns in early and late stages, matching the different effects of HGF. Certain bacteria were linked to inflammation and bone loss, and predicted inflammatory pathways varied with disease stage. These findings suggest HGF shapes bacterial communities in ways that may explain its stage-dependent impact on periodontitis.

**Abstract:**

Background: Periodontitis is a chronic disease triggered by disturbed oral microbiota. We have previously reported that hepatocyte growth factor (HGF) could mitigate early-stage experimental periodontitis but exacerbate the condition in its late stage. Here, we investigated the impact of HGF on the periodontal microbiome during periodontitis progression. Methods: We established ligation-induced periodontitis in wild-type (WT) mice and HGF high-expression transgenic (HGF-Tg) mice. We quantified the levels of IL-6 and TNF-α in periodontal tissues, as well as the serum concentrations of CTXI and PINP. Ligatures were collected on days 0, 7, and 28 after ligation for 16S rRNA sequencing and microbial analysis. Results: HGF significantly altered the diversity of ligatures during periodontitis. Interestingly, specific microbial genera, such as Lactobacillus, exhibited opposing trends between the two disease stages of HGF-Tg mice, aligning with the different effects of HGF on periodontitis progression. We also identified some taxa, such as Sphingomonas, associated with IL-6, TNF-α, CTXI, and PINP. The predicted inflammatory pathways (e.g., IL-17 signaling pathways) were enriched in HGF-Tg mice on day 28 but decreased on day 7. Conclusions: HGF exerted different influences on the microbiota of ligatures during early and late stages of periodontitis, which may account for the divergent effects of HGF on periodontitis progression.

## 1. Introduction

Periodontitis is a chronic inflammatory disease characterized by the progressive degradation of periodontal tissues [1]. It is widely accepted that periodontitis is instigated by oral microbial dysbiosis, characterized by distinct alterations in microbial structure, composition, and metabolic activities [2,3]. Specific pathogens and their virulence factors have been shown to promote the progression of periodontitis [4,5,6]. Clinical evidence and animal experiments have also revealed that targeting microbial imbalances can be an effective treatment for periodontitis [7,8,9]. Although the contribution of periodontitis-associated species cannot be ignored, the comprehensive interaction between the entire microbial community and the host response may play a more important role in the progression of periodontitis [2]. On the one hand, dysbiosis of the periodontal microbiome drives both local and systemic host responses that exacerbate inflammation and tissue degradation [10]. On the other hand, the specific oral microenvironment and host responses, influenced by the microbial community, may further create conditions conducive to the proliferation of periodontitis-associated pathogens and microbial imbalance [11,12]. Active management of inflammation led to the natural elimination of periodontal pathogens without the need for mechanical or antimicrobial treatments [13]. Overall, susceptibility to periodontitis and subsequent tissue damage is influenced by the interplay between bacteria and host response [2].

Multiple cytokines, such as human growth factors, have been reported to be involved in the interaction between the host response and microbiome alteration during periodontitis [14]. Current research has also highlighted hepatocyte growth factor (HGF), a pleiotropic cytokine, responsible for inflammatory and immune responses in various diseases [15]. Elevated levels of HGF in oral rinse and gingival cervical fluids (GCFs) were associated with the severity of periodontitis [16]. A recent study also demonstrated a negative correlation between serum HGF levels and the abundance of *Firmicutes* in gut microbiota [17]. However, direct mechanistic evidence from genetically modified models, such as HGF overexpression or knockout mice, remains lacking, and no prior research has specifically explored the role of HGF in shaping the oral microbiota.

Our preliminary findings revealed that HGF played a protective role in the early stage of experimental periodontitis but exacerbated the bone destruction and inflammation in the late phase [18]. Based on these observations, we hypothesize that HGF exerts stage-specific effects on periodontitis, which are associated with distinct changes in microbiota at different stages. This study aims to elucidate the impact of HGF on the microbiota of ligatures using ligature-induced periodontitis in wild-type (WT) and HGF high-expression transgenic (HGF-Tg) mice.

## 2. Materials and Methods

### 2.1. Animals

Twelve wild-type (WT) C57BL/6 (control, C) and twelve HGF high-expression transgenic (HGF-Tg, H) male mice aged 6 weeks were used in this study. Wild-type mice were purchased from Guangdong Experimental Animal Center (Guangzhou, China). The creation and genotyping of HGF-Tg mice were conducted in accordance with protocols previously established [18,19]. In brief, these mice exhibit elevated HGF expression in oral tissues [18]. The mice were housed under standardized conditions of humidity (50–60%) and temperature (20–24 °C) with a 12/12 h light/dark cycle. The Guangdong Huawei Testing Co., Ltd.’s (Guangzhou, China) Animal Research Ethics Committee approved all animal care and experimental protocols (Approval No. HWT-BG-117).

### 2.2. Ligature-Induced Periodontitis (LIP) Model

A ligature-induced periodontitis model was established by placing a 5-0 silk around the right maxillary second molar, following established procedures [18]. The mice were randomly divided into six groups: WT mice ligated for 0 days (C0); WT mice ligated for 7 days (C7); WT mice ligated for 28 days (C28); HGF-Tg mice ligated for 0 days (H0); HGF-Tg mice ligated for 7 days (H7); HGF-Tg mice ligated for 28 days (H28). Ligation for 7 days was classified as the early stage of periodontitis, and ligation for 28 days was defined as the late stage of periodontitis [20]. Ligatures were collected and stored at −80 °C under sterile conditions for use as previously described [10]. The periodontal tissues surrounding the teeth affected by periodontitis and the serum were collected and stored at −80 °C.

### 2.3. Protein Expression Analysis by ELISA

Interleukin-6 (IL-6) and tumor necrosis factor-α (TNF-α) levels in periodontal tissue around the teeth were assessed using mouse ELISA Kits following the manufacturer’s instructions (Cusabio, Wuhan, China). The levels of C-telopeptide of type I collagen (CTXI) and procollagen I N-terminal pro-peptide (PINP) in serum were determined using mouse ELISA Kits in accordance with the manufacturer’s guidelines (Elabscience, Wuhan, China). These measurements have been previously reported [18], and the data were used here to examine correlations with the oral microbiota composition.

### 2.4. 16S rRNA Sequencing, Bioinformatic, and Statistical Analysis

Following the sample collection of ligatures, DNA extraction was performed using E.Z.N.A.^®^ soil DNA Kit (Omega Bio-tek, Norcross, GA, USA), with subsequent quantification of DNA concentration and assessment of purity. PCR amplification of the V3–V4 regions of the 16S rRNA gene was then carried out using the previous primers on an ABI GeneAmp^®^ 9700 PCR thermocycler (ABI, Vernon, CA, USA), with optimization of PCR conditions for specificity and yield [21]. After library preparation, sequencing was performed on the Illumina MiSeq PE300 platform (Illumina, San Diego, CA, USA) using standard protocols provided by Majorbio Bio-Pharm Technology Co., Ltd. (Shanghai, China).

Bioinformatic microbiome of ligatures was analyzed on the Majorbio Cloud platform (http://cloud.majorbio.com, accessed on 20 February 2025) [22]. Data analysis encompassed quality control and trimming of raw reads, as well as classification of operational taxonomic units (OTUs) with a 97% sequence similarity level [23]. Subsequent to rarefaction, α-diversity indices were measured by Mothur v1.30.1, and β-diversity was visualized by principal coordinate analysis (PCoA) plots based on Bray–Curtis dissimilarity [24]. The linear discriminant analysis effect size (LEfSe) was employed to determine the significant taxa of bacteria among the different groups, with linear discriminant analysis (LDA) score > 3 and *p* < 0.05 [25]. Redundancy analysis (RDA) was applied to identify the relationship between clinical parameters and oral microbiota [26]. Spearman correlations were also conducted to delineate the relationship between taxa and environmental indices. PICRUSt2 analysis and STAMP software (version 2.1.0) facilitated the prediction and verification of distinct microbial pathways [27,28].

Statistical analyses were conducted using GraphPad Prism version 8.0 or R software (version 4.2.2). The Gaussian distribution of the data was evaluated with the Shapiro–Wilk test, while Levene’s test was used to assess the homogeneity of variances. Multigroup comparisons were performed using one-way ANOVA for normally distributed data and the Kruskal–Wallis test for non-normally distributed data. Depending on the data distribution, we employed either Student’s *t*-test or the Wilcoxon rank-sum test. *p*-values were adjusted using the Benjamini–Hochberg method. Results were expressed as the mean ± standard deviation (SD), with a *p*-value of less than 0.05 considered statistically significant.

## 3. Results

### 3.1. HGF Altered LIP Microbial Diversity During Periodontitis Development

**Figure 1:**
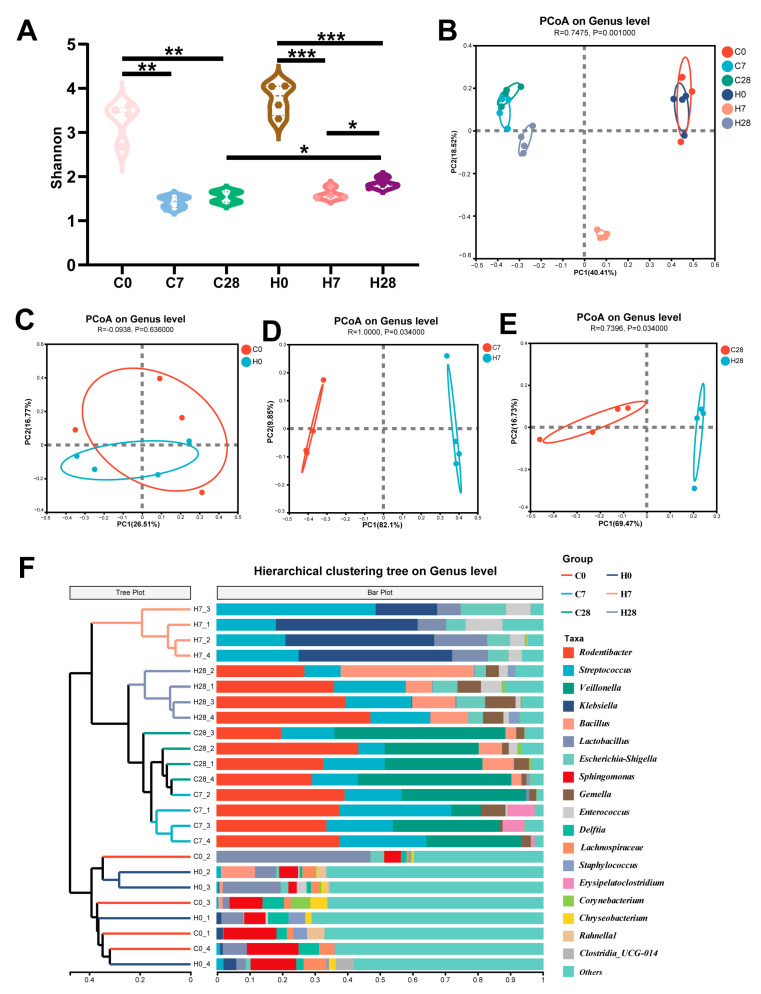
(**A**) Assessment of ligature microbiota α-diversity among WT mice (control, C) on day 0 (C0), day 7 (C7), and day 28 (C28) together with HGF-Tg mice (H) on day 0 (H0), day 7 (H7), and day 28 (H28). (**B**–**E**) Principal coordinate analysis (PCoA) plots illustrating the genus-level distribution, utilizing Bray–Curtis distance metrics. (**F**) Cluster analysis of all samples across six groups. *, *p* < 0.05; **, *p* < 0.01; ***, *p* < 0.001.

In both WT and HGF-Tg mice, α-diversity significantly decreased on days 7 and 28 compared to day 0 (*p* < 0.01), indicating a reduction in microbial diversity as periodontitis developed (Figure 1A). On days 0 and 7, the α-diversity was not different between the WT and HGF-Tg mice. However, HGF-Tg mice presented statistically higher α-diversity compared with WT mice on day 28 (*p* < 0.05) (Figure 1A). Differences between the LIP microbial construction of groups were analyzed by PCoA (β-diversity) and cluster analysis. Generally, the ligature microbial community of LIP groups on different time points tended to cluster separately at the genus level when compared with the controls (Figure 1B,F). It was also worth noting that the H7 group exhibited distinct segregation from other LIP groups according to the PCoA plot (Figure 1B). At baseline, the microbial compositions in ligature sites from HGF-Tg and WT mice were similar (*p* ≥ 0.05) (Figure 1C). Significant differences in ligature microbiota were observed between HGF-Tg and WT mice on day 7 (Figure 1D), as well as between the two groups on day 28 (*p* < 0.05) (Figure 1E).

### 3.2. HGF Contributed to Community Shifts in LIP During Periodontitis Progression

**Figure 2:**
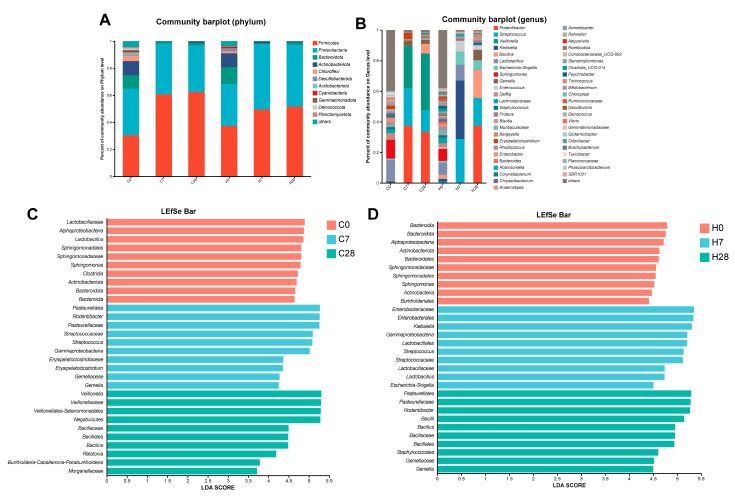
The relative abundance of microbiota in ligature sites at the phylum level (**A**) and genus level (**B**). (**C**,**D**) LEfSe analysis identified taxa with differential abundance, characterized by an LDA score exceeding 3.0 and a significance threshold of *p* < 0.05.

A stacked bar chart illustrated the difference in dominant taxa among all groups at the phylum and genus levels (Figure 2A,B). H7 group showed distinctly different composition compared with C7, C28, and H28 groups at the genus level (Figure 2B). The LEfSe analysis further revealed alterations in the oral microbiota of WT and HGF-Tg mice with periodontitis compared with controls (Figure 2C,D). On day 7, the proportions of *class_Gammaproteobacteria* and *genus_Streptococcus* were elevated in both WT and HGF-Tg mice. On day 28, an increased enrichment of *order_Bacillales* was observed in both WT and HGF-Tg mice (Figure 2C,D). The above results indicated a partially similar pattern in microbial shift during periodontitis progression in both WT and HGF-Tg mice.

**Figure 3:**
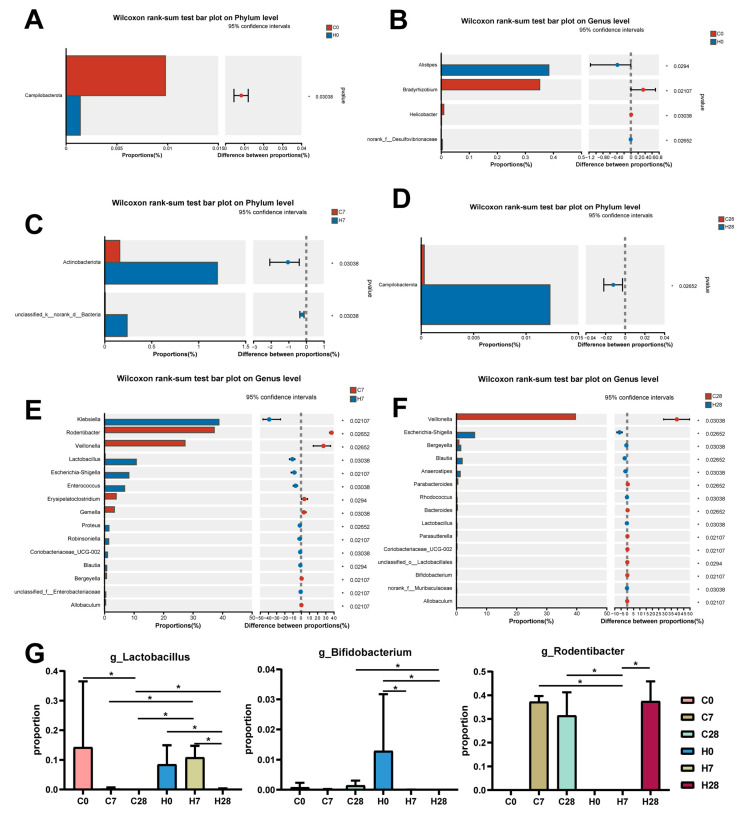
Comparison of proportions between the C0 and H0 groups at the phylum level (**A**) and genus level (**B**). Difference in microbial abundance between the C7 and H7 groups at the phylum level (**C**) and genus level (**E**). The differential taxa were identified between the C28 and H28 groups at the phylum level (**D**) and genus level (**F**). (**G**) The proportions of *g_Lactobacillus*, *g_Bifidobacterium*, and *g_Rodentibacter* among 6 groups. *, *p* < 0.05.

Then, we further demonstrated microbial taxa alteration between WT and HGF-Tg mice at different ligation time points. On day 0, *phylum_Campilobacterota*, *genus_Bradyrhizobium*, and *genus_Helicobacter* were more abundant in WT mice, while *genus_Alistipes* and *family_Desulfovibrionaceae* displayed a higher proportion in HGF-Tg mice (*p* < 0.05) (Figure 3A,B). On day 7, we found the abundance of *phylum_Actinobacteriota*, *genus_Klebsiella*, *genus_Lactobacillus*, *genus_Escherichia-Shigella*, *genus_Enterococcus,* and *genus_Coriobacteriaceae_UCG-002* were elevated, and *genus_Rodentibacter*, *genus_Veillonella*, *genus_Gemella* together with *genus_Bergeyella* exhibited lower prevalence in HGF-Tg mice (*p* < 0.05) (Figure 3C,E). On day 28, *phylum_Campilobacterota*, *genus_Bergeyella,* and *genus_Blautia* accounted for a higher proportion in HGF-Tg mice, contrasting with the enrichment of *genus_Veillonella*, *genus_Parabacteroides*, and *genus_Bacteroides* in WT mice (*p* < 0.05) (Figure 3D,F).

Notably, changes in certain flora, including *genus_Lactobacillus,* showed contrasting trends during the two disease periods of HGF-Tg mice, consistent with the different effects of HGF on periodontitis progression. Moreover, the abundance of the probiotic *genus_Bifidobacterium* was diminished in the H28 group compared with the C28 group. Concurrently, the proportion of pro-inflammatory *genus_Rodentibacter* in the C7, C28, and H28 groups was richer than the C0, H0, and H7 groups (Figure 3G).

### 3.3. Identification of LIP Microbiota Correlated with Inflammation and Bone Metabolism

**Figure 4:**
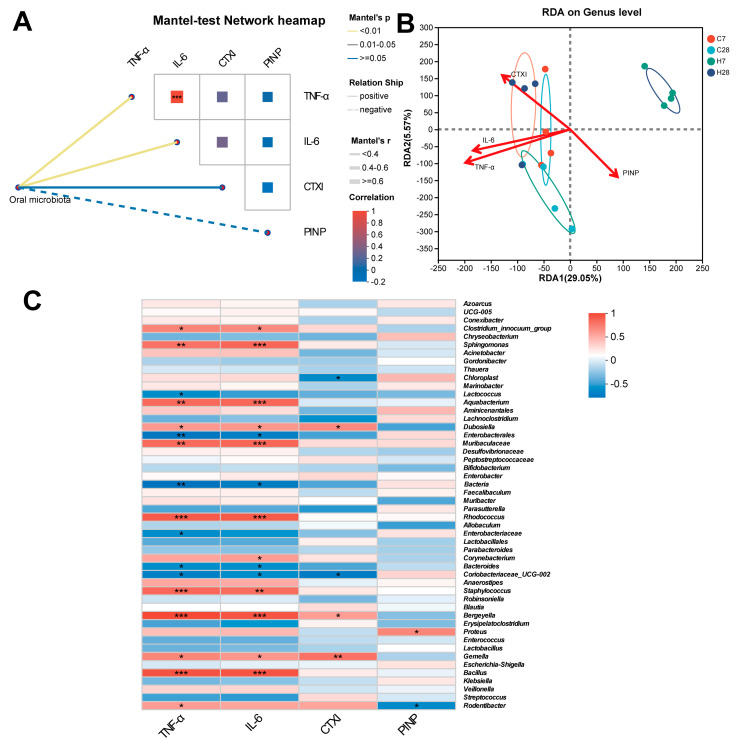
(**A**) A network heatmap generated from the Mantel test, showcasing the overarching relationships between the microbiota composition and inflammatory as well as bone metabolic markers. (**B**) RDA of microbial diversity and environmental indices. (**C**) Spearman correlation between genus and environmental indices was illustrated by a heatmap. *, *p* < 0.05; **, *p* < 0.01; ***, *p* < 0.001.

Typically, IL-6 and TNF-α were positively associated with bone destruction and inflammation of periodontitis; blood CTXI and PINP serve as markers of bone resorption and bone formation, respectively [20,29]. We previously found that HGF significantly decreased the levels of IL-6, TNF-α, and CTXI on day 7 but increased these indicators on day 28, and PINP showed no significant difference between WT and HGF-Tg mice [18]. Therefore, we examined the correlation between microbial community structure and these indices of laboratory tests. The Mantel test heatmap demonstrated IL-6 and TNF-α were positively associated with the community distance matrix (Figure 4A). RDA (Figure 4B, Table 1) revealed that IL-6, TNF-α, and CTXI showed a significantly positive correlation with the alterations in the C7, C28, and H28 groups, and a negative correlation with the microbial distribution of the H7 group (*p* < 0.05). We also employed the Spearman correlation test to investigate the association between these selected biomarkers and microbial taxa at the genus level. *Sphingomonas*, *Clostridium-innocuum-group*, *Aquabacterium*, *Enterobacterales*, *Rhodococcus*, *Staphylococcus*, *Bergeyella*, *Bacillus*, *Dubosiella*, and *Gemella* were positively associated with IL-6 and TNF-α. Conversely, *Bacteroides* and *Coriobacteriaceae* were negatively correlated with IL-6 and TNF-α. *Dubosiella*, *Bergeyella,* and *Gemella* were positively associated with CTXI, and *Proteus* were positively associated with PINP (Figure 4C).

### 3.4. HGF Shifted Microbial Function During Periodontitis Progression

**Figure 5:**
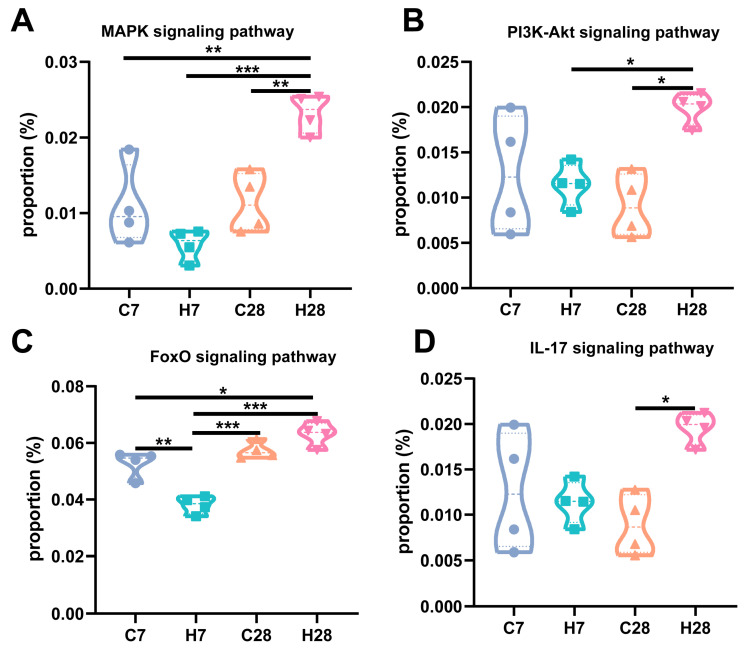
Comparative analysis of 4 pro-inflammatory PICRUSt-predicted pathways, including MAPK (**A**), PI3K-Akt (**B**), FoxO (**C**), and IL-17 (**D**) signaling pathways, was shown using violin plots. *, *p* < 0.05; **, *p* < 0.01; ***, *p* < 0.001.

To investigate the potential effects of HGF on the microbiota biological pathways of LIP, the abundance of functional categories was predicted using PICRUSt2 analysis. Our prior findings indicated that HGF reduced the expression of IL-6, TNF-α, and IL-17 on day 7, but enhanced these indices on day 28 [18]. This led us to hypothesize a connection between these alterations and the microbiota at ligature sites. As shown in Figure 5, the richness of inflammatory pathways, including mitogen-activated protein kinase (MAPK), phosphatidylinositol 3-kinase/protein kinase B (PI3K/Akt), forkhead box O (FoxO), and interleukin-17 (IL-17) signaling pathway, was higher in the H28 group than the C28 group. In addition, HGF also diminished the abundance of the FoxO signaling pathway at the early stage of periodontitis.

## 4. Discussion

In this study, we examined the ligature microbiota of HGF-Tg mice during the early and late stages of experimental periodontitis. Our findings revealed that HGF significantly altered microbial diversity and composition, which were linked to inflammation and bone metabolism during periodontitis. The changes in certain bacterial taxa and predicted inflammatory pathways showed different trends between the two stages, consistent with the different impact of HGF on periodontitis. These results suggested that the tissue destruction may be associated with a disease-oriented microbial shift affected by HGF.

Our study demonstrated that periodontitis reduced α-diversity in both WT and HGF-Tg mice, and HGF elevated α-diversity in the late stage of periodontitis. Reduced diversity, reflecting a single and unstable microbial community, was identified as a feature associated with the loss of commensals and the accumulation of pathogens [30]. A previous study also verified that the microbial structure was significantly shifted from control to periodontitis at all time points according to β-diversity [31], which was consistent with our result. Notably, the β-diversity exhibited clear spatial segregation between WT and HGF-Tg mice. These results suggested the distribution and composition of ligature microbiota may be partially influenced by HGF.

Further analysis identified specific microorganisms implicated in periodontitis. We observed that *Bifidobacterium* was less prevalent in the H28 group than in the C28 group. A randomized clinical trial displayed that *Bifidobacterium* enhanced additional clinical, microbiological, and immunological benefits in the treatment of periodontitis [32]. We also found that *Lactobacillus* was more or less prevalent in the C7, C28, and H28 groups than in the controls and the H7 group, and *Lactobacillus* served as a prebiotic beneficial for the treatment of periodontitis [8]. The underlying mechanisms mainly included its anti-inflammatory responses and suppression of periodontal pathogens [33]. *Rodentibater*, which participated in the development of experimental periodontitis [31], was also significantly more abundant in the C7, C28, and H28 groups but declined in the H7 group. This may be one of the reasons why the H7 group showed less bone damage and inflammation compared with other LIP groups.

It has been widely recognized that inflammation and bone destruction can be influenced by microbiota [34]. Therefore, we performed a correlation analysis to identify a specific genus linked with inflammation and bone metabolism in our study. Among them, the periodontitis-associated genus *Rodentibater* was positively correlated with TNF-α but inversely associated with PINP, further indicating that Rodentibater may be involved in inflammation and bone metabolism during periodontitis. Moreover, *Sphingomonas*, another microorganism positively associated with IL-6 and TNF-α in our study, was identified as more abundant in the gut of patients with colitis-associated cancer [35] and in an inflammation-associated animal model [36]. *Sphingomonas paucimobilis* (a species belonging to *Sphingomonas*) was the most frequently isolated subgingival non-oral Gram-negative bacterium from periodontitis [9]. We observed IL-6 and TNF-α were also positively related to *Clostridium-innocuum-group*, which was classified as a risk factor for hypertension, with IL-1R2 (an inflammation-associated receptor) being identified as a significant mediator [37]. The above evidence suggests that *Sphingomonas* and *the Clostridium-innocuum group* might participate in the inflammation of periodontitis. On the contrary, *Bacteroides* might exert an anti-inflammatory effect according to our analysis. *Bacteroides* is widely known as a dominant butyrate-producing bacterium in the intestine, and butyrate plays an anti-inflammatory role in periodontitis [38]. However, *Bacteroides* may act as an infecting organism to enhance damage to tissue [39]. Therefore, the precise causal relationship between these taxa and periodontitis is required for more sufficient and rigorous verification.

Our previous study demonstrated that HGF decreased the expression of pro-inflammatory cytokines IL-6, TNF-α, and IL-17 on day 7 but elevated these indices on day 28 compared with WT mice [18]. Considering oral microbial function involved in inflammation, we utilized PICRUSt2 analysis to depict the different microbial pathways modified by HGF. Intriguingly, MAPK, PI3K-Akt, FoxO, and IL-17 signaling pathways were activated in the H28 group but suppressed in the H7 group. It has been widely recognized that FoxO proteins, which are modulated by the MAPK and PI3K-Akt pathways, play a crucial role in regulating the transcription of IL-6 and TNF-α [40]. G. Calissi et al. also revealed that the FoxO signaling pathway was involved in Th17 differentiation and IL-17 secretion [41]. IL-17 is regarded as an inducer of osteoclast differentiation via upregulating TRAF6 expression and RANKL/OPG ratio, thus enhancing bone loss in periodontitis [42,43]. Interestingly, our prior findings also indicated that HGF exerted divergent regulatory effects on the IL-17/RANKL/OPG axis in different stages of periodontitis [18]. The above evidence demonstrates that HGF may contribute to the inflammatory response and bone metabolism at different stages of periodontitis by affecting microbial functions.

Nevertheless, this study had several limitations. We examined the ligature microbiota, which did not fully capture the complexity of the periodontal microbiota. Although PICRUSt2 provides useful insights into potential microbial functions, it relies on reference genome annotations and does not directly measure gene expression or biological activity. The specific mechanism by which periodontitis is altered by microbes in the presence of HGF remains unclear. Possible explanations include indirect regulation through modulation of host immune responses, epithelial barrier integrity, or inflammatory mediators, which in turn reshape the microbial community. Future studies integrating metatranscriptomics, metabolomics, and host–microbe interaction models will be crucial to elucidate how HGF mechanistically influences the oral microbiome.

## 5. Conclusions

Overall, our findings revealed stepwise shifts in the ligature-associated microbiome during the development of periodontitis. While microbial dysbiosis was a common feature, the influence of HGF varied according to the disease stage. Specifically, HGF appeared to mitigate dysbiosis in the early stage but exacerbate microbial imbalance in the late stage. These findings not only provide new insight into the complex interactions between host factors and microbial ecology during periodontal disease progression but also suggest that therapeutic strategies targeting HGF–microbiota interactions may need to be stage-tailored to achieve optimal outcomes.

## Figures and Tables

**Table 1:** The RDA between oral microbiota and selected indicators.

	RDA1	RDA2	R^2^	* p * Value
TNF-α	−0.9987	−0.0515	0.4289	0.034
IL-6	−0.9992	0.0404	0.3657	0.045
CTXI	−0.8209	0.571	0.4613	0.013
PINP	0.7857	−0.6186	0.2793	0.104

## Data Availability

The raw 16S rRNA sequencing data have been submitted to NCBI under the SRA database [PRJNA1088630]. All authors have approved the data in this study for publication.

## Abbreviations

The following abbreviations are used in this manuscript:

HGF	Hepatocyte Growth Factor
WT	Wild Type
HGF-Tg	Hepatocyte Growth Factor High-Expression Transgenic
TNF-α	Tumor Necrosis Factor-α
IL-6	Interleukin-6
IL-17	Interleukin-17
LIP	Ligature-induced periodontitis
CTXI	C-terminal telopeptide of type I collagen
PINP	N-terminal pro-peptide of type I procollagen
MAPK	mitogen-activated protein kinase
PI3K/Akt	Phosphatidylinositol-3-kinase/protein kinase B
FoxO	Forkhead box O
TRAF6	TNF receptor-associated factor 6
RANKL	Receptor Activator of Nuclear Factor-κ B Ligand
OPG	Osteoclastogenesis inhibitory factor
OTU	Operational Taxonomic Units
PCoA	Principal coordinate analysis
LDA	Linear Discriminant Analysis
LEfSe	Linear Discriminant Analysis Effect Size
PICRUSt	Phylogenetic Investigation of Communities by Reconstruction of Unobserved States
RDA	Redundancy Analysis

## References

[1] Kassebaum N.J., Bernabé E., Dahiya M., Bhandari B., Murray C.J.L., Marcenes W. (2014). Global burden of severe periodontitis in 1990–2010: A systematic review and meta-regression. J. Dent. Res..

[2] Curtis M.A., Diaz P.I., Van Dyke T.E. (2020). The role of the microbiota in periodontal disease. Periodontology 2000.

[3] Chen Z., Zhong Y., Chen L., Liu W., Lin C., Chen Y., Wang X. (2025). HGF Aggravated Periodontitis-Associated Gut Barrier and Microbial Dysfunction: Implications for Oral–Gut Axis Regulation. Biology.

[4] Blasco-Baque V., Garidou L., Pomié C., Escoula Q., Loubieres P., Le Gall-David S., Lemaitre M., Nicolas S., Klopp P., Waget A. (2017). Periodontitis induced by Porphyromonas gingivalis drives periodontal microbiota dysbiosis and insulin resistance via an impaired adaptive immune response. Gut.

[5] Pan C., Liu J., Wang H., Song J., Tan L., Zhao H. (2017). Porphyromonas gingivalis can invade periodontal ligament stem cells. BMC Microbiol..

[6] Wang Y., Wang L., Sun T., Shen S., Li Z., Ma X., Gu X., Zhang X., Peng A., Xu X. (2023). Study of the inflammatory activating process in the early stage of Fusobacterium nucleatum infected PDLSCs. Int. J. Oral Sci..

[7] Hajishengallis G., Liang S., Payne M.A., Hashim A., Jotwani R., Eskan M.A., McIntosh M.L., Alsam A., Kirkwood K.L., Lambris J.D. (2011). Low-abundance biofilm species orchestrates inflammatory periodontal disease through the commensal microbiota and complement. Cell Host Microbe.

[8] Homayouni Rad A., Pourjafar H., Mirzakhani E. (2023). A comprehensive review of the application of probiotics and postbiotics in oral health. Front. Cell. Infect. Microbiol..

[9] van Winkelhoff A.J., Rurenga P., Wekema-Mulder G.J., Singadji Z.M., Rams T.E. (2016). Non-oral gram-negative facultative rods in chronic periodontitis microbiota. Microb. Pathog..

[10] Ribeiro A.A., Jiao Y., Girnary M., Alves T., Chen L., Farrell A., Wu D., Teles F., Inohara N., Swanson K.V. (2022). Oral biofilm dysbiosis during experimental periodontitis. Mol. Oral Microbiol..

[11] Dutzan N., Kajikawa T., Abusleme L., Greenwell-Wild T., Zuazo C.E., Ikeuchi T., Brenchley L., Abe T., Hurabielle C., Martin D. (2018). A dysbiotic microbiome triggers T_H_17 cells to mediate oral mucosal immunopathology in mice and humans. Sci. Transl. Med..

[12] Kittaka M., Yoshimoto T., Schlosser C., Kajiya M., Kurihara H., Reichenberger E.J., Ueki Y. (2020). Microbe-Dependent Exacerbated Alveolar Bone Destruction in Heterozygous Cherubism Mice. JBMR Plus.

[13] Lee C.-T., Teles R., Kantarci A., Chen T., McCafferty J., Starr J.R., Brito L.C.N., Paster B.J., Van Dyke T.E. (2016). Resolvin E1 Reverses Experimental Periodontitis and Dysbiosis. J. Immunol..

[14] Afacan B., Keleş Yücel Z.P., Paşali Ç., Atmaca İlhan H., Köse T., Emingil G. (2020). Effect of non-surgical periodontal treatment on gingival crevicular fluid hypoxia inducible factor-1 alpha, vascular endothelial growth factor and tumor necrosis factor-alpha levels in generalized aggressive periodontitis patients. J. Periodontol..

[15] Molnarfi N., Benkhoucha M., Funakoshi H., Nakamura T., Lalive P.H. (2015). Hepatocyte growth factor: A regulator of inflammation and autoimmunity. Autoimmun. Rev..

[16] Nagaraja C., Pradeep A.R. (2007). Hepatocyte growth factor levels in gingival crevicular fluid in health, disease, and after treatment. J. Periodontol..

[17] Alamri T., Alkhaldy A.A., Gauthaman K., Pushparaj P.N., Moulay M., Mirza A.A., Azhar E.I., Barnawi S., Papadopoulou G., Karamitros T. (2022). Growth factors in relation to obesity, food habits, and microbiota among healthy Saudis: Preliminary results. Eur. Rev. Med. Pharmacol. Sci..

[18] Zhao X., Liu W., Wu Z., He X., Tang Y., He Q., Lin C., Chen Y., Luo G., Yu T. (2024). Hepatocyte growth factor is protective in early stage but bone-destructive in late stage of experimental periodontitis. J. Periodontal Res..

[19] Wang X., Yan L., Tang Y., He X., Zhao X., Liu W., Wu Z., Luo G. (2022). Anti-inflammatory effect of HGF responses to oral traumatic ulcers using an HGF-Tg mouse model. Exp. Anim..

[20] de Molon R.S., Park C.H., Jin Q., Sugai J., Cirelli J.A. (2018). Characterization of ligature-induced experimental periodontitis. Microsc. Res. Tech..

[21] Liu C., Zhao D., Ma W., Guo Y., Wang A., Wang Q., Lee D.-J. (2016). Denitrifying sulfide removal process on high-salinity wastewaters in the presence of Halomonas sp.. Appl. Microbiol. Biotechnol..

[22] Ren Y., Yu G., Shi C., Liu L., Guo Q., Han C., Zhang D., Zhang L., Liu B., Gao H. (2022). Majorbio Cloud: A one-stop, comprehensive bioinformatic platform for multiomics analyses. iMeta.

[23] Edgar R.C. (2013). UPARSE: Highly accurate OTU sequences from microbial amplicon reads. Nat. Methods.

[24] Schloss P.D., Westcott S.L., Ryabin T., Hall J.R., Hartmann M., Hollister E.B., Lesniewski R.A., Oakley B.B., Parks D.H., Robinson C.J. (2009). Introducing mothur: Open-source, platform-independent, community-supported software for describing and comparing microbial communities. Appl. Environ. Microbiol..

[25] Segata N., Izard J., Waldron L., Gevers D., Miropolsky L., Garrett W.S., Huttenhower C. (2011). Metagenomic biomarker discovery and explanation. Genome Biol..

[26] Oksanen J., Kindt R., Legendre P., O’hara B., Stevens M., Oksanen M., Suggests M. (2007). The Vegan Package: Community Ecology Package.

[27] Douglas G.M., Maffei V.J., Zaneveld J.R., Yurgel S.N., Brown J.R., Taylor C.M., Huttenhower C., Langille M.G.I. (2020). PICRUSt2 for prediction of metagenome functions. Nat. Biotechnol..

[28] Parks D.H., Tyson G.W., Hugenholtz P., Beiko R.G. (2014). STAMP: Statistical analysis of taxonomic and functional profiles. Bioinformatics.

[29] Eastell R., Szulc P. (2017). Use of bone turnover markers in postmenopausal osteoporosis. Lancet Diabetes Endocrinol..

[30] Petersen C., Round J.L. (2014). Defining dysbiosis and its influence on host immunity and disease. Cell. Microbiol..

[31] Arce M., Endo N., Dutzan N., Abusleme L. (2022). A reappraisal of microbiome dysbiosis during experimental periodontitis. Mol. Oral Microbiol..

[32] Invernici M.M., Salvador S.L., Silva P.H.F., Soares M.S.M., Casarin R., Palioto D.B., Souza S.L.S., Taba M., Novaes A.B., Furlaneto F.A.C. (2018). Effects of Bifidobacterium probiotic on the treatment of chronic periodontitis: A randomized clinical trial. J. Clin. Periodontol..

[33] İnce G., Gürsoy H., İpçi Ş.D., Cakar G., Emekli-Alturfan E., Yılmaz S. (2015). Clinical and Biochemical Evaluation of Lozenges Containing Lactobacillus reuteri as an Adjunct to Non-Surgical Periodontal Therapy in Chronic Periodontitis. J. Periodontol..

[34] Ebersole J., Kirakodu S., Chen J., Nagarajan R., Gonzalez O.A. (2020). Oral Microbiome and Gingival Transcriptome Profiles of Ligature-Induced Periodontitis. J. Dent. Res..

[35] Richard M.L., Liguori G., Lamas B., Brandi G., da Costa G., Hoffmann T.W., Pierluigi Di Simone M., Calabrese C., Poggioli G., Langella P. (2018). Mucosa-associated microbiota dysbiosis in colitis associated cancer. Gut Microbes.

[36] Gao W.-T., Liu J.-X., Wang D.-H., Sun H.-J., Zhang X.-Y. (2023). Melatonin reduced colon inflammation but had no effect on energy metabolism in ageing Mongolian gerbils (*Meriones unguiculatus*). Comp. Biochem. Physiol. C Toxicol. Pharmacol..

[37] Zhao S., Zhang J., Ding F., Sun S. (2023). Relationships among gut microbes, the interleukin family, and hypertension: A mediation Mendelian randomization study. Front. Nutr..

[38] Wu L., Luo Z., Chen Y., Yan Z., Fu J., Jiang Y., Xu J., Liu Y. (2023). Butyrate Inhibits Dendritic Cell Activation and Alleviates Periodontitis. J. Dent. Res..

[39] Wexler H.M. (2007). Bacteroides: The good, the bad, and the nitty-gritty. Clin. Microbiol. Rev..

[40] Kim M.E., Kim D.H., Lee J.S. (2022). FoxO Transcription Factors: Applicability as a Novel Immune Cell Regulators and Therapeutic Targets in Oxidative Stress-Related Diseases. Int. J. Mol. Sci..

[41] Hedrick S.M., Hess Michelini R., Doedens A.L., Goldrath A.W., Stone E.L. (2012). FOXO transcription factors throughout T cell biology. Nat. Rev. Immunol..

[42] Xiao E., Mattos M., Vieira G.H.A., Chen S., Corrêa J.D., Wu Y., Albiero M.L., Bittinger K., Graves D.T. (2017). Diabetes Enhances IL-17 Expression and Alters the Oral Microbiome to Increase Its Pathogenicity. Cell Host Microbe.

[43] Chen Z., Weng J., Du X., Ji R., Yang X., Yang Y., Ma M. (2025). Quaternized chitosan/glycyrrhizic acid co-decorated titanium with enhanced antimicrobial, immunomodulatory, and osteogenic properties for dental implant applications. Carbohydr. Polym..

